# Mouse Models for Food Allergies: Where Do We Stand?

**DOI:** 10.3390/cells8060546

**Published:** 2019-06-06

**Authors:** Stefan Schülke, Melanie Albrecht

**Affiliations:** Paul-Ehrlich-Institut, Vice President´s Research Group 1: Molecular Allergology, 63225 Langen (Hesse), Germany; Melanie.Albrecht@pei.de

**Keywords:** food allergy, mouse model, adjuvant, humanized mice

## Abstract

Food allergies are a steadily increasing health and economic problem. Immunologically, food allergic reactions are caused by pathological, allergen-specific Th2 responses resulting in IgE-mediated mast cell degranulation and associated inflammatory reactions. Clinically, food allergies are characterized by local inflammation of the mouth mucosa, the face, the throat, the gastrointestinal tract, are frequently paralleled by skin reactions, and can result in life-threatening anaphylactic reactions. To better understand food allergies and establish novel treatment options, mouse models are indispensable. This review discusses the available mouse food allergy models, dividing them into four categories: (1) adjuvant-free mouse models, (2) mouse models relying on adjuvants to establish allergen-specific Th2 responses, (3) mouse models using genetically-modified mouse strains to allow for easier sensitization, and (4) humanized mouse models in which different immunodeficient mouse strains are reconstituted with human immune or stem cells to investigate humanized immune responses. While most of the available mouse models can reproducibly portray the immunological parameters of food allergy (Th2 immune responses, IgE production and mast cell activation/expansion), so far, the recreation of the clinical parameters has proven more difficult. Therefore, up to now none of the available mouse models can reproduce the complete human pathology.

## 1. Introduction

### 1.1. Relevance of Food Allergies 

With a prevalence of 8% in children and 4% in adults, food allergies are a growing health and economic problem [1,2,3]. Here, not only the frequency of food allergies is increasing, but also their severity. In line with this, 40% of food allergic children were reported to suffer from severe type reactions (e.g., shortness of breath, shock type reactions) upon contact with the respective allergen [2]. When analyzing data from the USA, food allergies cause 50,000 visits to the emergency room per year [4] with 150 to 200 fatal reactions [2] and are responsible for approximately 500 million USD in both direct (e.g., hospital care and doctor visits) and indirect (e.g., loss of work force) costs [5]. In terms of allergen sources, 90% of food allergic reactions are caused by either egg, milk, wheat, soy, peanuts, tree nuts, shellfish, or fish (the so-called “red flag foods“) [6,7].

### 1.2. Pathomechanism of Food Allergy

**Figure 1:**
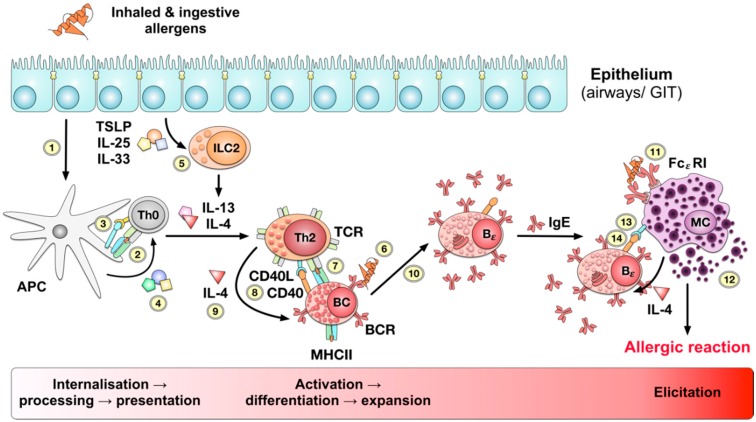
Pathomechanism of type I allergy. Uptake or penetration of allergen molecules via the respiratory or gastrointestinal epithelia (1) results in uptake, processing and presentation of allergen-derived peptides by antigen presenting cells (APCs, (2)). Activated APCs express co-stimulatory signals (3) and secrete cytokines (4) that promote the differentiation of naïve antigen-specific T cells into effector cells. In case of allergic reactions naive allergen-specific Th0 cells differentiate into Th2 cells orchestrated by the cytokines IL-13 and IL-4 produced by innate like lymphocytes type II (ILC2). The respective ILC2s are in turn activated by the cytokines thymic stromal lymphopoietin (TSLP), IL-25, and IL-33 secreted by either stressed or damaged epithelial cells (5). The activated allergen-specific Th2 cells in turn activate allergen-specific naïve B cells that have specifically recognized the allergen via their B cell receptor (BCR, (6)), taken up, processed, and presented the allergen to the T cell receptor (TCR) of the activated Th2 cell (7). The Th2 cell subsequently authorizes the activation and differentiation of the allergen-specific B cell via CD40 and CD40 ligand (CD40L) signaling (8) and secretion of the Th2-promoting cytokine IL-4 (9). Together, these signals allow for the differentiation of the allergen-specific B cell into an IgE-producing plasma cell (10). The allergen-specific IgE antibodies produced in turn by the activated plasma cell bind to the high affinity IgE receptor FcεRI on the surface of mast cells, resulting in the sensitization of these cells (11). Upon second contact with the allergen the allergic reaction is triggered by the cross-linking of the surface bound IgE antibodies resulting in the degranulation of the mast cell associated with the release of large amounts of pro-inflammatory mediators (12). The activated mast cells in a positive feedback loop drive further activation of allergen-specific B cells by the production of IL-4 and providing CD40L-mediated co-stimulatory signals (13) to CD40 on the B cell surface (14).

Immunologically, food allergies are caused by allergic type I reactions according to Coombs and Gell. In allergic patients, uptake of the allergen via epithelia, e.g., in the gastrointestinal tract, results in the uptake, processing, and presentation of allergen-derived peptides on the surface of antigen presenting cells (APCs, Figure 1) [8,9].

Naive, allergen-specific T cells activated by these APCs in turn differentiate into T helper 2 (Th2) cells, directed by cytokines (e.g., interleukin 25 (IL-25) or thymic stromal lymphopoietin (TSLP)) or damage associated molecular patterns (DAMPs) released from damaged epithelial cells (e.g., IL-33), which subsequently trigger the local activation of innate like lymphocytes type II cells (ILC2s) [10,11]. Upon activation, these ILC2s provide cytokines IL-13 and IL-4 driving Th2 activation and differentiation (Figure 1) [12].

Once activated, allergen-specific Th2 cells enable activation, differentiation, and isotype switching of allergen-specific B cells towards IgE-producing plasma cells. The produced allergen-specific IgE antibodies subsequently bind to the high affinity IgE receptor FcεRI on the surface of mast cells and basophils, resulting in their sensitization towards the allergen (Figure 1) [13]. Interestingly, IgE-binding to CD23 (FcεRII), albeit with considerable lesser affinity, modulates IgE synthesis and might thus be an interesting target for therapeutic interventions (IgE-binding to FcεRI and FcεRII reviewed in [14]).

Upon second contact with the allergen, cross-linking of surface-bound IgE antibodies results in mast cell and eosinophil activation and local release of histamine, cytokines, proteases, hormones, and other pro-inflammatory mediators in a process called degranulation which then causes the allergic inflammation and the associated symptoms (Figure 1).

Therefore, the allergic type I reaction is characterized by a Th2-mediated IgE production resulting in mast cell activation and expansion [13]. In affected food allergic patients, these processes lead to a local inflammation of the mouth mucosa, the face, the throat, and are often paralleled by skin reactions [15]. Furthermore, food allergic patients suffer from abdominal pain, nausea, diarrhea, or vomiting caused by local inflammation of the gastrointestinal tract [16]. In addition, if initiated, systemic mast cell activation can result in acute life-threatening anaphylactic reactions.

## 2. Factors Contributing to the Development of Food Allergy

**Figure 2:**
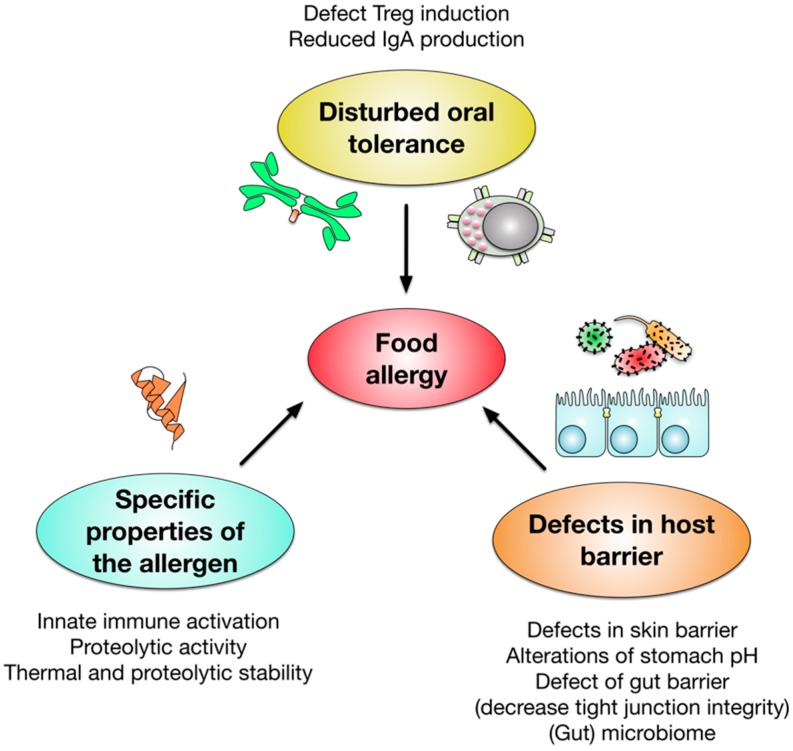
Factors contributing to the development of food allergy. The published literature suggests, that both a disruption of regulatory T cell (Treg)- and immune globulin A (IgA)-mediated oral tolerance and defects in the barrier function of skin and gut contribute to the development of food allergy. Moreover, alterations in stomach pH and the gut and skin microbiome were shown to influence allergic sensitization. In addition, intrinsic properties of the allergen molecules such as innate immune activation, proteolytic activity, or increased thermal and proteolytic stability contribute to allergic sensitization.

Multiple factors were reported to contribute to the complex development of food allergies (see Figure 2). In food allergic patients we observe a disrupted oral tolerance development characterized by defects in the induction of regulatory T cells and production of neutralizing, allergen-specific IgA antibodies (reviewed in [13,17]). In addition, defects in the epithelial barrier, both in the skin and the gut, as well as alterations in stomach pH, reportedly promote allergy development (reviewed in [13]). Additionally, we are just starting to understand that the microbiome can also contribute to allergic sensitization and germ-free mice show a remarkable Th2 bias [18,19,20].

In addition, the specific properties of the allergens themselves contribute to their allergenicity. Here, increased sensitizing properties were attributed to allergen molecules with either intrinsic immune activating properties (reviewed in [21]), proteolytic activity [22], or increased stability towards heat and proteases [22,23,24].

One recently published study illustrates how for example the microbiome may influence allergy development. In this study Feehley and coauthors reconstituted gnotobiotic C3/HeN mice with feces from either healthy or cow’s milk allergic patients and subsequently sensitized the animals to beta lactoglobulin using cholera toxin as an adjuvant [25]. Finally, both groups were challenged twice within 30 min orally with 100 mg beta lactoglobulin [25]. Here, only the microbiome of healthy patients was able to efficiently protect mice against allergic sensitization, while animals reconstituted with the feces of allergic patients showed a strong temperature drop and an increased production of allergen-specific IgE antibodies [25].

In additional experiments the authors were able to show that the protective effect of the microbiome of non-allergic donors could be substituted by the clostridia species *Anaerostipes caccae* [25]. Reconstitution of gnotobiotic mice with *Anaerostipes caccae* before allergic sensitization efficiently prevented the observed hypothermia, and suppressed allergen-specific IgG1 and IgE production, mast cell activation, as well as production of the Th2 cytokines IL-4 and IL-13 [25].

In summary, food allergy is caused by a combination of genetic as well as environmental factors influencing risk and mode of disease manifestation, a complex topic which is addressed in many other publications (for example reviewed in [26,27]).

## 3. Advantages of Animal Models for Food Allergy Research

Currently, food allergies are treated with a combination of allergen avoidance and symptomatic treatment with epinephrine (rescue medication) and anti-inflammatory drugs (e.g., steroids or anti-histamines) [28,29]. Allergen-specific immunotherapy (AIT), the only disease-modifying treatment, is currently not established due to the severe side effects observed during the first clinical trials using subcutaneous injection of allergens [30]. Nevertheless, more recent clinical research shows promising results with administration of food allergens via the oral route, which might result in possible treatment options for food allergies in the future [31,32].

Therefore, there is an urgent need to establish new treatment options for food allergic patients. For the establishment of novel treatment options, animal models are indispensable, since they allow us:

(1) to investigate the immune responses underlying the allergic pathology,
(2) to establish and compare the allergenic potency of candidate molecules, and
(3) to evaluate the potency and safety of novel therapeutic options and vaccines derived from these findings in vivo without endangering the lives and health of the patients.

Noteworthy, animal models for allergenicity prediction are not covered in this review.

Here, the majority of the published animal models for food allergy use either swine, dog, rat, or mouse as model species [33]. This review focusses on mouse models of food allergy, which make up the biggest portion of the available food allergy models.

There is no naturally occurring allergic mouse; thus, the induction of sensitization and allergic reactions in mice will always be an artificial process. Apart from the numerous similarities between mice and men with regards to the immunological mechanisms underlying sensitization and clinical symptoms of food allergy, certain differences exist that should be considered when assessing results obtained from mouse models in terms of translation to human disease. One example of these disparities is the induction of anaphylactic reactions in an IgE-independent way via IgG and Fcγ receptors, which is described in mice, but does not play a prominent role in human allergic anaphylactic reactions [34,35,36]. Other differences such as—but not limited to—unequal expression profiles of FcεRI receptors on cell types (in mice restricted to basophils and mast cells, in humans expressed on other cell types as well), diverging effects of IL-13 on B-cell isotype switching to IgE (only in human) and diverging mast cell profiles are addressed in other publications, and are therefore not included in this review [37,38].

However, mouse model systems have certain key advantages such as short generation times, small size, relatively low costs of maintenance, relative ease of genetic manipulation with established methods, and the availability of many different well characterized genetic backgrounds as well as deficient/transgenic strains [18].

## 4. General Characteristics of the Models

Among the mouse models of food allergy, one can distinguish between (1) models that just investigate immune responses towards the applied allergens, such as induction of Th2 responses, IgE and IgG1 production, or mast cell activation and (2) models that also try to recreate the clinical symptoms of the food allergic reaction such as local inflammation in the gut, the mouth, and face, anaphylactic reactions, and behavioral changes (reduced activity). Models that only investigate immune responses towards the allergens are mainly used for risk assessment of potentially allergenic proteins, while mouse models additionally reflecting the clinical pathology associated with food allergy can in theory be used to better understand the underlying pathomechanisms and to evaluate novel treatment options.

In case of models only investigating allergen-specific IgE induction, it is noteworthy that clinically irrelevant IgE sensitizations are frequently observed in non-allergic patients and therefore have only limited prognostic value [39].

**Figure 3:**
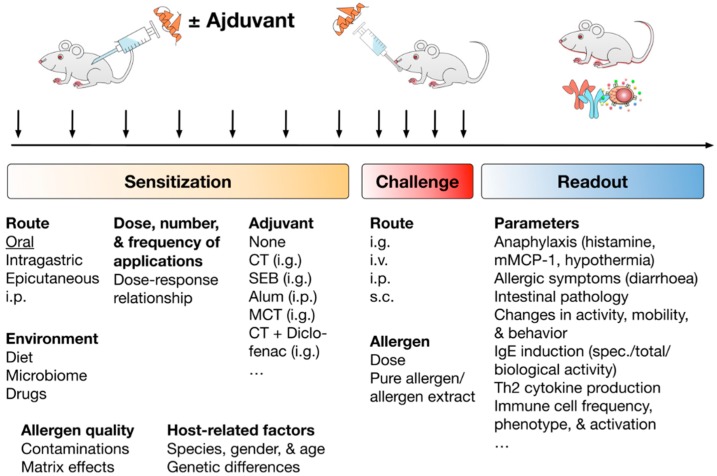
Experimental setup, factors contributing to the successful establishment, and readout parameters typically investigated in the published mouse food allergy models. Successful establishment of allergic sensitization in mouse food allergy models was shown to depend on the route of allergen application, dose, frequency, and number of applications, as well as the used adjuvant. In addition, environmental factors such as diet, microbiome of the used animals, and the eventual usage of drugs during the sensitization, allergen quality with respect to contaminations with either other proteins or endotoxins, matrix effects of the applied allergen extract, and host-related factors such as used mouse strain, gender, and age of the used animals, but also genetic differences were shown to be critical factors in allergic sensitization. Subsequently to successful allergic sensitization animals are challenged with either purified allergens or allergen-containing allergen extracts to elicit the allergic reaction. The main differences in the published mouse food allergy models are observed for the routes of allergen application, allergen dose, and usage of either purified allergens or allergen-enriched natural extracts of the respective food source. Finally, the induced allergic reactions are evaluated according to certain readout parameters. These readout parameters are divided into clinical parameters of the allergic reaction (e.g., induced anaphylactic reactions, intestinal allergic symptoms, as well as changes in animal activity, mobility and behavior) and parameters that characterize the underlying immunologic reactions (production of allergen-specific IgG1 and IgE, biological activity of the induced IgE antibodies, induction of Th2 responses and associated cytokine production, differences in immune cell phenotype, frequency, and activation). Abbreviations: i.p.: intraperitoneal, i.g.: intragastric, i.v. intravenous, s.c.: subcutaneous, CT: cholera toxin, SEB: *Staphylococcus* enterotoxin B, Alum: aluminum hydroxide, MCT: medium chain triglycerides.

Typically, the experimental setup of the published food allergy mouse models can be divided into three parts: (1) sensitization towards the investigated allergen, (2) the challenge with the allergen (usually following after a certain rest period) to induce the allergic reaction, and (3) the evaluation of the established allergic reaction using the chosen clinical and immunological readout (see Figure 3).

The biggest variations among the published mouse models can be found in the sensitization phase. Here, the chosen route of application, dose, number, and frequency of applications, as well as the choice of adjuvant, can critically influence sensitization success. Although every allergen behaves differently, in general, lower allergen amounts were repeatedly shown to have a higher sensitizing potential than corresponding higher allergen doses [40]. Moreover, the oral route of sensitization should be preferred if the effects of digestion and the interaction of the allergens with the epithelia are to be simulated [18].

In addition, environmental factors such as diet, microbiome, and a potential treatment of the animals with drugs during the experiment as well as the quality of the used allergens in respect to protein folding, contamination with other proteins or endotoxins, and matrix effects should be considered. Finally, host factors such as the chosen mouse strain, sex, and age of the animals, as well as genetic differences between the animals may also influence the sensitization process [18].

In line with this, Brandt and colleagues reported, that the commonly used mouse strains BALB/c and C57BL/6 strongly differ in their ability to be sensitized towards ovalbumin (Ova) [41]. When both mouse strains were sensitized against Ova by two-time i.p. injection with aluminum hydroxide, only BALB/c mice developed diarrhea upon oral challenge with 50 mg of Ova [41]. Arumugam and colleagues further compared BALB/c mice to 129ScEvBrd mice [42]. Here, both strains were sensitized by a single s.c. injection of 50 µg Ova in aluminum hydroxide and challenged orally 14 days later with 50 mg Ova [42]. Interestingly, in this direct comparison, 129ScEvBrd mice exhibited a significantly stronger temperature drop which the authors correlated to a higher number of mast cells in the tissues of the 129ScEvBrd mice compared to BALB/c animals [42].

With respect to the challenge the most frequently varied parameters are route of application, allergen dose, and source of the allergen (e.g., purified allergen vs. enriched extracts) (see Figure 3).

Finally, the readout of the induced allergic reactions is divided into clinical and immunological parameters: Clinically, the development and severity of the induced anaphylaxis, clinical symptoms of the gastrointestinal inflammation, changes in animal activity, mobility, and behavior are investigated (see Figure 3). Immunologic parameters usually comprise the induction of Th2 responses, production of either total or allergen-specific IgG1 and IgE antibodies, their respective biological activity, the induced mast cell activation, as well as the changes in the frequency, phenotype, or activation of other relevant immune cell types.

## 5. Usage of Adjuvants in Food Allergy Mouse Models

The uptake of a food allergen usually results in its processing and presentation on the surface of APCs via MHC II molecules. Since most allergens do not encode for an intrinsic danger signal, the allergen uptake does not result in the activation of the respective APC and naive allergen-specific T cells are preferentially differentiated into regulatory T cells [43]. This naturally occurring oral tolerance towards orally applied allergens needs to be broken for the successful establishment of mouse food allergy models.

For this, different adjuvants are currently used. Here, the activation of e.g., pattern recognition receptors by such adjuvants promotes APC activation and leads to the expression of co-stimulatory molecules and production of cytokines. Hereby, the adjuvants used for the establishment of food allergy models favor the differentiation of naive allergen-specific T cells into Th2 cells. In addition, adjuvant-induced APC activation triggers its migration to the draining lymph nodes where immune responses can be efficiently initiated [18].

The most frequently used adjuvants for the establishment of food allergy models are cholera toxin (CT, [17,44,45,46]), staphylococcus enterotoxin B (SEB, [47]), aluminium hydroxide (alum, [41,48,49]), and lipopolysaccharide (LPS, [50]).

Oral application of allergens with CT results in a robust production of allergen-specific IgE antibodies, which was shown to depend on the ability of CT to activate cyclic adenosine monophosphate (cAMP) production in CD11b^+^ dendritic cells [51,52]. In contrast to this, SEB is a bacterial superantigen that induces antigen-unspecific T cell activation by crosslinking of the T cell receptor (TCR) and MHC molecules [47]. Alum and LPS both are classical immune activators: while Alum crystals activate the NLRP3 inflammasome [53], LPS is a ligand for “Toll”-like receptor 4 (TLR4), triggering APC activation via mitogen-activated protein kinase (MAPK)-and nuclear factor “kappa-light-enhancer” of activated B cells (NFκB)-signaling [54].

Many of the published mouse food allergy models use cholera toxin to break oral tolerance towards co-applied allergens [16,40,45,46,51,55,56]. For example, Sun et al. reported a mouse model in which C57BL/6 mice were sensitized against peanut protein by four times i.p. injection with cholera toxin [57]. Upon i.p. challenge with 5 mg peanut protein the sensitized animals developed a pronounced temperature drop, local swelling in the area of nose and mouth, and displayed a reduced activity [57]. Serologically, these effects were paralleled by the production of allergen-specific IgG1 and IgE antibodies that were further increased with every additional sensitization, as well as the systemic release of the mast cell mediators histamine and cysteinyl leukotriene [57]. In this model intraperitoneal challenge with the allergen resulted in an early mast cell activation, followed by an infiltration of neutrophils, lymphocytes, and eosinophils into the peritoneum [57].

However, the usage of adjuvants has both advantages and disadvantages: While adjuvants allow to break oral tolerance, enabling oral sensitizations and both faster and stronger immune responses to otherwise low-immunogenic molecules, the strong immune activating effects of the adjuvants often make it hard to determine the contribution of the respective food allergen to the observed immune responses. In addition, the usage of strong adjuvants can also result in the induction of Th2 responses by nonallergenic proteins [58].

In contrast to this, interpretation of the obtained results is easier for adjuvant-free sensitization models since here the observed immune responses are exclusively induced by the allergen. Moreover, adjuvant-free sensitization models are usually a lower burden for the animals. However, it was suggested that the usually applied transdermal or systemic sensitization routes to achieve adjuvant-free most models may not reflect the physiological routes of sensitization (reviewed in [58]). In addition, the above described readout parameters are usually less pronounced in adjuvant-free sensitization models compared to mouse models using adjuvants for sensitization.

**Figure 4:**
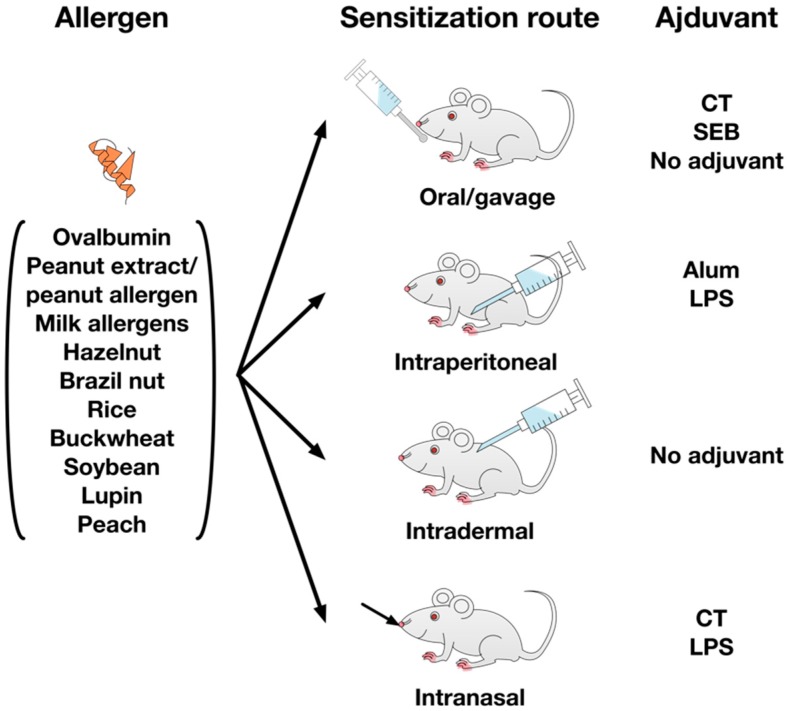
Allergens and adjuvant strategies described in the literature to establish food allergy models. The published mouse food allergy models employ a variety of different food allergens with hen’s egg, milk, and nut allergens making up the majority of the used allergen sources. To achieve allergic sensitization, the different allergens are either applied orally with cholera toxin (CT), *Staphylococcus* enterotoxin B (SEB), or adjuvant-free, intraperitoneally with aluminum hydroxide (Alum) or lipopolysaccharide (LPS), intradermally without adjuvant, or intranasally together with CT and LPS.

The published mouse models describe several different allergens, that are either given orally using cholera toxin, staphylococcus enterotoxin B, or without adjuvant, intraperitoneally with LPS or aluminum hydroxide, transdermally without adjuvant, or intranasally using cholera toxin or LPS as adjuvants (see Figure 4).

## 6. Types of Mouse Food Allergy Models

The available literature contains a steadily increasing number of published mouse food allergy models (734 hits in the PubMed database as of April 2019). Therefore, this review cannot describe all the available literature.

**Figure 5:**
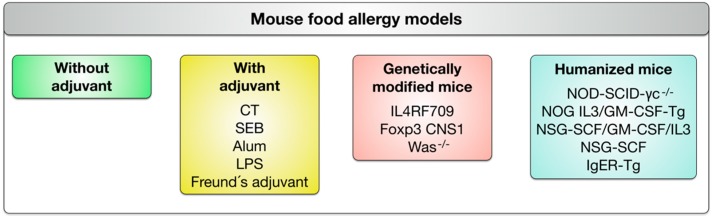
Classification of mouse food allergy models. The available mouse models are divided into four categories: (1) adjuvant-free mouse models, (2) mouse models relying on adjuvants to establish allergen-specific Th2 responses, (3) mouse models using genetically-modified mouse strains to allow for easier sensitization, and (4) humanized mouse models in which different immunodeficient mouse strains are reconstituted with human immune or stem cells to investigate humanized immune responses.

In order to structure the different mouse food allergy models we suggest to divide them into four categories: (1) adjuvant-free mouse models, (2) mouse models relying on different adjuvants to establish allergen-specific Th2 responses, (3) mouse models using genetically-modified mouse strains to allow for easier sensitization, and (4) humanized mouse models in which different immunodeficient mouse strains are reconstituted with human immune or stem cells to investigate human immune responses in these animals (see Figure 5).

In the following section we will present some selected examples for each category (in addition summarized in Table 1).

### 6.1. Adjuvant-Free Models

Adjuvants are not always required to achieve allergic sensitization. That even oral sensitization without adjuvant is possible was demonstrated for example by Proust and colleagues. In their study C3H/HeJ mice were sensitized to peanut by a single oral application of 80 mg peanut extract and sensitized animals were challenged 4 to 21 days later by intraperitoneal application of peanut extract [59]. This experimental approach resulted in the production of peanut specific IgE antibodies, local mast cell activation in the skin, as well as mast cell-mediated anaphylactic reactions 7 to 14 days after sensitization [59].

Interestingly, epidemiological data suggest, that sensitization to food allergens may also occur through skin exposure to peanut-containing oil if the barrier function of the respective skin area is disrupted, e.g., by inflammation, while oral exposure to peanut early in life rather results in tolerance development [60,61,62]. In line with these observations, defects in epithelial barrier function by loss of function mutations of filaggrin were correlated with higher risks of, among others, atopic dermatitis and peanut allergies [63,64].

Therefore, adjuvant-free sensitization models frequently make use of the transdermal sensitization route, resulting in allergic sensitization [65,66,67]. In one such study, BALB/c mice were transdermally sensitized to the egg allergen Ova by repeated tape stripping, and were subsequently challenged orally with 100 mg of Ova [68]. Interestingly, in this model the repeated tape stripping and the associated damage of the skin resulted in a release of the DAMP cytokine IL-33 [68]. Mechanistically, it was shown that the release of IL-33 was essential for the induction of allergic reactions, since, for example, a blockade of either IL-33 production or IL-33 receptor ST2 were sufficient to both suppress the observed Ova-induced allergic reactions and to reduce mouse mast cell protease 1 (mMCP1) secretion [68]. Hence, this study shows that allergic immune responses induced by transdermal sensitization can bypass oral tolerance development. At least in this model, the inflammatory signals necessary to break allergen-specific tolerance are supplied by damaging of the skin.

### 6.2. Adjuvanted Mouse Food Allergy Models

The number of available mouse food allergy models using adjuvants to achieve allergic sensitization is extensive. Here, we will point out some exemplary studies highlighting interesting features.

In 2000 Li and co-authors published an influential mouse food allergy model. They sensitized C3H/HeJ mice two times weekly with either 5 or 25 mg peanut protein plus 10 µg cholera toxin orally and challenged the animals 21 days later with a single dose of 10 mg peanut extract orally [40]. With their study they could show, that the use of cholera toxin as an adjuvant efficiently induces allergen-specific Th2 responses since the animals showed a robust peanut-specific IgE production, mast cell activation, and plasma histamine release compared to non-immunized animals [40]. In their hands only the lower allergen dose (5 mg extract), but not the higher dose (25 mg extract) was able to successfully induce allergic sensitization [40]. Moreover, Li et al. defined a symptom score for the evaluation of the induced allergic reactions (Table 2) which has proven very useful and thus has been frequently used for other food allergy models [17,49,50,69,70,71].

Our group established a mouse model of allergic enteritis [72]. Here, BALB/c mice are sensitized against Ova by two times i.p. injection of the allergen together with alum. One week after the last sensitization, the animals were challenged by continuous feeding of Ova-containing food pellets for a period of seven days [72]. In sensitized animals, this protocol results in a strong weight loss and temperature drop, as well as a pronounced diarrhea [72].

That LPS can be used as adjuvant to achieve allergic sensitization is demonstrated by the work of Rodriguez and co-authors. In this study, BALB/c mice were sensitized six times weekly with the major peach allergen Pru p 3 and LPS intranasally and subsequently challenged one week after the last sensitization by i.p. application of the allergen [50]. The challenge with Pru p 3 resulted in anaphylactic reactions characterized by a temperature drop, as well as inactivity and increased respiratory rates in LPS plus Pru p 3 sensitized animals [50]. Serologically, these effects were paralleled by an increased production of Pru p 3-specific IgE and IgG1 antibodies compared for example to animals sensitized with only the allergen [50]. In line with these results, an increased production of the cytokines IL-4 and IFN-γ was observed from splenocytes isolated from animals that were sensitized with LPS plus Pru p 3, while the production of the anti-inflammatory cytokine IL-10 was reduced [50].

Finally, Tanaka et al. showed that mouse models can even be used to simulated more rare types of allergies such as exercise-induced anaphylaxis. In their model Tanaka sensitized B10.A mice 6 times weekly with the wheat protein gliadin and aluminum hydroxide, subsequently challenged the animals with 10 mg gliadin by oral administration, and forced the animals to undergo 30 min of strenuous physical exercise on a treadmill (15 m/min, 20% gradient) [48]. Here, sensitization resulted in the production of gliadin-specific IgE antibodies while challenge provoked a marked temperature drop [48]. Interestingly, sensitized and challenged animals showed significantly lower activity levels upon exposure to strenuous exercise, suggesting the successful induction of exercise induced anaphylaxis in these animals [48].

### 6.3. Mouse Models Using Genetically-Modified Mouse Strains

Several mouse food allergy models make use of genetically-modified mouse strains. Here, the introduced genetic changes aim to facilitate an allergic, Th2-biassed sensitization by either disrupting the differentiation and function of Tregs or directly facilitating the induction of Th2 responses.

One example of such a mouse strain are IL-4RaF079 mice. In these mice the inhibitory ITIM motif of the IL-4 receptor alpha chain was inactivated via knock in mutagenesis by replacing the tyrosine residue at position 709 with a phenylalanine [83]. Although this change does not reflect any known human mutation it is prototypical for different IL-4Ra polymorphisms observed in atopic individuals [83]. By pairing this mutated IL-4R alpha chain with either the common gamma chain or the IL-13R alpha 1 chain, a constitutively active receptor is formed that mediates Th2 responses via binding of IL-4 or IL-13 to either the type I or type II IL-4R [73,83].

When initially characterizing these animals, Tachdijan and colleagues reported naive T cells from these animals differentiated into Th2 cells to produce significantly higher levels of the Th2 promoting cytokine IL-4 compared to equally treated wildtype cells [83]. Moreover, if IL-4 was added to naive B cells isolated from the IL-4RaF709 mice an increased phosphorylation level of the Th2 differentiation factor STAT-6 was detected [83].

This Th2-polarizing milieu in the IL-4RaF709 mice resulted in both a significantly increased basal IgE production in naive IL-4RaF709 animals as well as increased allergen-specific IgE levels if the animals were sensitized with Ova plus CT. Moreover, the IL-4RaF709 mice could be sensitized to Ova by repeated oral application of the allergen without adjuvant in a way that subsequent oral challenge with Ova resulted in a strong temperature drop and the occurrence of diarrhea [73]. These clinal symptoms were paralleled by a systemic mast cell activation as well as a production of ovalbumin-specific IgE antibodies [73].

Two more genetically-modified mouse models describe a facilitated sensitization towards food allergens by disrupting Treg differentiation and function: the CNS1^−/−^ mice and the WAS^−/−^ mice. Non-coding DNA sequence 1 (CNS1) is an intronic enhancer at the Foxp3 locus [84]. It was found to contain a TGF-β–NFAT response element (driving TGF-β-mediated Foxp3 expression) and to have a prominent function in the generation of peripherally induced Tregs (iTregs) in gut-associated lymphoid tissues [74,84]. Interestingly, Foxp3 CNS1 was found to be dispensable for either the differentiation of thymus-derived Tregs or maintenance of Foxp3 expression in differentiated Tregs [74,84].

In line with the important function of iTregs in the establishment and maintenance of oral tolerance in the gastrointestinal tract [85], the defect of iTregs in Foxp3 CNS1^−/−^ mice was reported to result in the spontaneous development of Th2-biased responses characterized by enhanced production of IL-4, IL-5, and IL-13 by GATA binding protein 3 (GATA3)^+^CD4^+^ T cells (especially in the mesenteric lymph nodes, Peyer’s patches and intestinal lamina propria), constitutively increased serum levels of IgE and IgA specific for both autoantigens (derived from the small and large intestine, pancreas), as well as food antigens, and alterations in the gut microbiome (decreases in the ratio of Firmicutes to Bacteroidetes) [74]. Interestingly, Th1 and Th17 responses were unchanged by this Foxp3 CNS1-mediated defect in iTreg induction and no multiorgan autoimmune responses were observed [74].

This selective Th2 bias in Foxp3 CNS1^−/−^ mice caused distinct Th2-mediated pathologies at both the gastrointestinal tract and the lung characteristic of both allergic inflammation and asthma [74]. The gastrointestinal pathology comprised a decrease in body weight likely caused by gastritis and plasmacytic enteritis with increased frequencies of plasma cells in the intestinal lamina propria and crypt abscesses [74]. Overall, the published results suggest, that the Th2-promoting immunologic state in Foxp3 CNS1^−/−^ mice can be used to facilitate allergen specific sensitization in food allergy models.

Finally, mice deficient for the Wiskott-Aldrich syndrome protein (WASP) were shown to exhibit spontaneous IgE production towards food proteins. In human patients, mutations in the cytosolic WASP result in the Wiskott-Aldrich syndrome (WAS), an X-linked primary immunodeficiency that is characterized by a progressive decline in T-cell numbers, failure to produce antibodies to polysaccharide and protein antigens, low levels of serum IgM, and elevated levels of IgE resulting in recurrent infections, thrombocytopenia, gastrointestinal bleeding, eczema, and a predisposition to lymphocyte-mediated malignancies [76,86]. WASP is only expressed in cells of hematopoietic origin where it is involved in TCR-mediated signal transduction to the cytoskeleton [87].

While WAS^−/−^ mice, in contrast to WAS patients, have normal levels and production of antibodies, WAS^−/−^ mice show similarities to WAS patients too: e.g. defects in T-cell receptor–induced proliferation and aberrant regulation of the actin cytoskeleton, as well as reduced numbers of Tregs, resulting in lymphopenia [86]. In line with observations from human WAS patients WAS^−/−^ mice develop T cell dependent, severe progressive colitis characterized by both infiltration of neutrophils and lymphocytes and enhanced levels of IFN-γ, IL-4, and IL-13 by six months of age (50% fatality rate) [75]. Despite the observed induction of Th1 and Th2 cytokines, only neutralization of IL-4, but not IFN-y, was able to abrogate the development of colitis, suggesting the observed intestinal inflammation to be Th2-dependent [75].

Accordingly, WAS^−/−^ mice were reported to develop spontaneous allergic sensitization to common food allergens, most pronounced for wheat and soy, which was paralleled by intestinal mast cell expansion [76]. In this model Th2-mediated intestinal inflammation was even further enhanced when mice were conditionally deleted for WASP in Foxp3^+^ Tregs, suggesting that WASP-deficiency drives Th2 responses by interfering with normal Treg function [76].

Lexmond and colleagues demonstrated, that compared to classical Ova/alum sensitization, WAS^−/−^ mice orally sensitized to Ova without adjuvant (7 times 5 mg Ova in 5-day intervals) developed significantly lower Ova-specific IgE levels [77]. However, upon oral challenge with 50 mg Ova 79% of WAS^−/−^ mice exhibited symptoms of systemic intestinal anaphylaxis, with elevated serum levels of mMCPT1 and mortality rates greater than 50%, showing that the low-levels of food specific IgE antibodies observed in WAS^−/−^ mice were sufficient to trigger severe allergic reactions. Therefore, WAS^−/−^ mice mimic both the polysensitization and low-level but highly bioactive IgE production frequently observed in human food allergic patients [77]. In light of these results WAS^−/−^ mice may be an interesting model system to study spontaneous oral sensitization towards food allergens.

### 6.4. Humanized Mouse Models

In order to overcome some of the disparities between murine and human immunology (for more information see chapter 3 “Advantages of animal models for food allergy research”), humanized mouse models offer the unique possibility to investigate human immune responses in mice. This can be achieved by either modifying certain parts of the murine immune system into “humanized” components or by replacing the whole murine immune system with human cells generating (immunologically) fully humanized mice.

One example for modification of mouse cell populations is the generation of mice bearing the human FcεRI on the surface of DCs generated by Sallmann et al. [88]. As Sallmann and colleagues were interested in the role of IgE/FcεRI activity on DCs in the context of delayed type pulmonary allergic inflammation and–as stated above–mouse DCs do not express FcεRI on their surface, Sallmann and colleagues generated mice that express the α-chain of human FcεRI in a CD11c-specific manner, forming chimeric, active FcεRI on murine DCs in vivo [88]. These mice were used by Platzer and colleagues in a murine model of experimental food allergy in order to analyze the contribution of IgE/FcεRI binding on DCs in mucosal inflammation [78]. They demonstrated that FcεRI-humanized mice after being subjected twice to intraperitoneal sensitization (Ova 100 µg) and subsequent challenge with 50 mg Ova via gavage on alternating days for three to six times, presented with less gastrointestinal inflammation compared to wildtype control mice, although sensitization levels were comparable in both mouse strains. Thus, using a mouse model, with only a slight change in receptor expression towards a more “humanized” expression pattern, Platzer et al. could reveal a beforehand unappreciated regulatory function of IgE-signaling on DCs in allergic mucosal inflammation [78].

For the establishment of fully humanized mouse models immunodeficient mouse strains are engrafted with either human immune or stem cells and (after successful engraftment) subjected to experimental allergy models. Commonly used for efficient generation of humanized models are NOD-SCID-γc^−/−^ mice, who are non-obese diabetic (NOD), homozygous for severe combined immunodeficiency mutation (SCID) and lack the common gamma-chain (γc; IL-2Rγ) functionality; they have no mouse T and B cells, lack residual NK cell activity, and present with high engraftment rates of human cells (NOG mice: [80]; NSG mice: [89]).

Such mice were applied to a model of gut inflammation by Weigmann and colleagues, by interperitoneal injection of 2 × 10^7^ Peripheral blood mononuclear cells (PBMC) from allergic donors with high allergen-specific IgE values in combination with the respective allergen, followed by an intraperitoneal boost with allergen 8 days after cell transfer. Gut inflammation elicited by means of either rectal or oral allergen challenge three weeks after initial cell transfer was monitored via high-resolution video mini-endoscopy, histology, and further in vitro experiments [79]. Gastrointestinal inflammation was highly allergen-specific as Weigmann et al. could demonstrate that challenge by application of either saline or not-cross-reactive allergens did not result in inflammatory responses [79].

In order to improve development of human granulocytes and mast cells in humanized mice, Ito et al. generated NOG mice ubiquitously expressing human IL-3 and human granulocyte-macrophage colony-stimulating factor (GM-CSF) (NOG IL-3/GM-Tg mice) and applied 4–5 × 10^4^ human umbilical cord blood-derived CD34^+^ hematopoietic stem cells (HSC) intravenously into NOG IL-3/GM-Tg 24 h after irradiation [90]. 11 weeks after HSC engraftment, a significant increase in human total myeloid cells (CD33^+^), granulocytes (CD66b^+^), and monocytes (CD14^+^) in peripheral blood in comparison to NOG mice could be detected. Moreover, Ito and colleagues identified human eosinophils, basophils, neutrophils, and mast cells 18 weeks after transplantation and demonstrated that human basophils and mast cells from those mice upregulated activation markers upon stimulation in a similar way as observed with cells from human PBMCs [90]. Moreover, functionality of human mast cells in NOG IL-3/GM-Tg mice was confirmed by human IgE-mediated passive cutaneous anaphylaxis reactions, rendering this humanized mouse model an interesting tool for food allergy research.

In a more recent study published by Burton et al. immunodeficient NSG mice, that additionally express a human stem cell factor (SCF) transgene facilitating the development of human mast cells in these animals, were reconstituted with human CD34^+^ cord blood stem cells [81]. 16 weeks after reconstitution, the animals were sensitized against peanut by repeated oral application of peanut butter (8 times weekly 22.5 mg peanut butter i.g.) and finally challenged with a single oral dose of 350 mg peanut butter [81].

16 weeks after reconstitution, the major adaptive human cell types (Th1- and Th2-effector cells, Foxp3^+^CD127^low^CD25^+^ Tregs, and CD19^+^HLA-DR^+^ B cells) were detected in spleen and jejunum of the engrafted animals [81]. These human immune cells proved to be functional, since this experimental procedure resulted in the production of human IgE antibodies activating human mast cells (which were generated within these animals) [81]. Together, these processes resulted in the triggering of anaphylactic reactions, showing that such humanized mouse models can be used to recreate complex human immune responses [81].

Taken together, sophisticated approaches for modeling food allergy in the context of a humanized immune system in mice at hand, enable us to study allergic mechanisms as well as the impact of novel treatment options as is exemplary shown in a publication by Pagovich et al. They tested the efficacy of an adeno-associated virus (AAV)-based transfer of anti-human IgE as possible vaccine in a humanized mouse model of peanut allergy [82]. In detail, NSG mice were engrafted with PBMCs from peanut allergic individuals or control subjects and subsequently sensitized by 5 weekly intraperitoneal injections with peanut extract, followed by 4 weekly intragastric challenges with 300 µg peanut extract, leading to induction of human peanut specific IgE levels and allergic symptoms in peanut allergic mice. A single application of the AAV-based vaccine (10^11^ genome copies) three weeks before human cell transplantation led to noticeable generation of serum anti-human IgE levels and reduced clinical symptoms after peanut challenge [82]. The humanized form of this model allowed for direct comparison of this experimental vaccine with anti-human IgE (omalizumab), a treatment option approved for use in humans, and resulted in data suggesting a prolonged protective effect of the AAV-based vaccine compared to one injection with omalizumab [82].

Although humanized mouse models of allergic diseases still have their limitations (e.g., efficacy of engraftment, finite time window of possible studies, due to onset of graft versus host disease [91]), they add another excellent option to study food allergies.

## 7. Summary and Conclusions

The field of mouse food allergy models has made great progress in recent decades. Studies have shown that rodents can be sensitized to food allergens either with or without the use of additional adjuvants, resulting in allergic responses upon re-exposure to the respective allergens that reflect allergic responses observed in human patients [33]. Although not as commonly used as mice, other animal models not covered in this review like guinea pig, rat, or neonatal swine might prove useful for certain research applications, as they reflect different aspects of allergic diseases than mice; e.g., piglets are outbred, thus might be better situated simulating genetic diversity as inbred mice strains [92,93,94,95].

**Figure 6:**
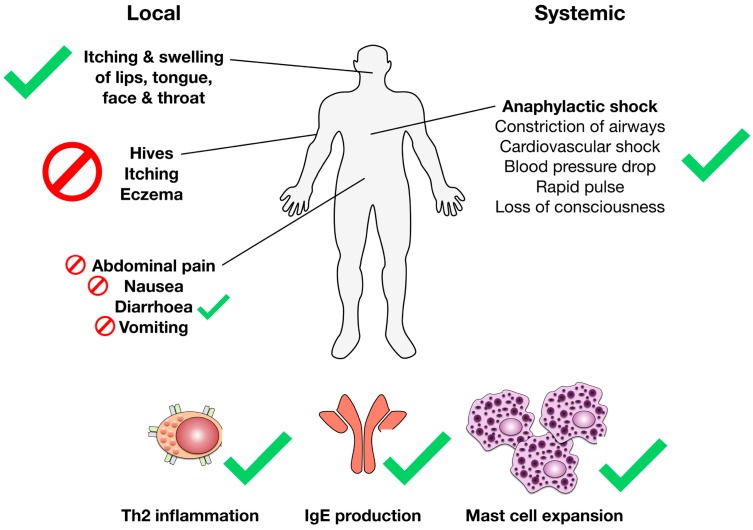
Reproduction of the immunological and clinical parameters associated with food allergic reactions by the available mouse food allergy models. Human immunological and clinical food allergy parameters that can be recreated by the available mouse food allergy models (anaphylactic shock and the associated parameters and symptoms, itching and swelling of lips, tongue, face, and throat, diarrhea caused by allergic intestinal inflammation, and the immunological parameters of the allergic Th2 reaction (Th2 inflammation, IgE production, as well mast cell activation, degranulation, and expansion)) are marked with green checkmarks, parameters that have up to now proven difficult to reproduce in mice (the often with food allergic reactions associated skin reactions (hives, itching, and eczema), abdominal pain, and nausea) are marked with red stop signs. Please note that mice are anatomically unable to vomit.

Today most of the available models are able to routinely reproduce the immunological parameters of the food allergic reaction such as allergen-specific Th2 responses, IgE production, as well as mast cell activation and expansion (Figure 6). Here, it is noteworthy to point out that the frequency of allergen-specific antibodies upon usage of adjuvants in mouse models is often significantly higher than the frequencies usually observed in allergic patients. For example, Kanagaratham and co-authors reported frequencies of allergen-specific IgE antibodies of up to 80% in the mouse when using alum as an adjuvant, while allergen-specific IgE frequencies in allergic patients to a single food allergen were reported to be only approximately 0.1 to 15% [13].

When looking at the clinical parameters of food allergic reactions that are described for the available mouse food allergy models, the models are still incomplete. While anaphylactic reactions (which, in the mouse are often additionally mediated via IgG1 antibodies), local inflammation in the area of the mouth, throat, and face, as well as diarrhea can be relatively consistently induced by different mouse models, skin reactions (resulting in itching and ruffed fur) which are commonly associated with food allergic reactions, as well as other local symptoms of the gastrointestinal inflammation (nausea, abdominal pain, and vomiting (anatomically not possible in mice)), can so far not be reproduced or monitored in objective and quantitative ways by the available models (Figure 6). Therefore, while none of the available models is able to fully re-create the complete human pathology, the copiousness and diversity of available food allergy mouse models allows for careful selection of the most suitable model with regard to the scientific question to be answered.

## Figures and Tables

**Table 1:** Summary of the experimental mouse food allergy models discussed in this article. Abbreviations: mMCP1: mouse mast cell protease 1, Alum: aluminum hydroxide, CNS1: non-coding DNA sequence 1, WAS(P): Wiskott-Aldrich syndrome (protein), GATA3: GATA binding protein 3, PBMC: peripheral blood mononuclear cells.

**Adjuvant-Free Models**					
**Reference**	**Mouse Strain**	**Sensitization**	**Challenge**	**Read-Out**	**Comments**
[59]	C3H/HeJ	1 × 80 mg peanut extract, i.g.	1 × 30 mg peanut extract, i.p.	Production of peanut specific IgE antibodies Local mast cell activation in the skin Mast cell-mediated anaphylactic reactions (temperature drop, reduced breathing rate, increased mMCP-1 release)	
[68]	BALB/c	Three one-week cycles of tape stripping each followed by application of 100 µg Ova onto the skin	1 × 100 mg of Ova, i.g.	IL-33 release in the skin after tape stripping Anaphylactic reactions (temperature drop, mast cell expansion, systemic release of mMCP1, Ova-specific IgE production)	Skin damage induced by repeated tape stripping triggers release of IL-33 IL-33 essential for the induction of allergic reactions
**Adjuvanted Mouse Food Allergy Models**					
**Reference**	**Mouse Strain**	**Sensitization**	**Challenge**	**Read-Out**	**Comments**
[25]	Gnotobiotic C3H/HeN	20 mg β-lactoglobulin + 10 µg cholera toxin, i.g.	2 × 100 mg β-lactoglobulin, i.g. 30 min apart	Temperature drop Increased production of allergen-specific IgG1 and IgE antibodies Mast cell activation Production of IL-4 and IL-13	Before sensitization, gnotobiotic mice were reconstituted with feces from either healthy or food allergic patients
[41]	BALB/c vs. C57BL/6	2 × 50 µg Ova + 1 mg Alum, i.p.	1 × 50 mg Ova i.g.	Acute diarrhea with increased intestinal permeability, eosinophilia, and mastocytosis only in BALB/c mice	Effects shown to be mast cell-dependent
[42]	BALB/c vs. 129ScEvBrd	1 × 50 µg Ova + 2 mg Alum, s.c.	1 × 50 mg Ova, i.g.	129ScEvBrd mice with significantly stronger temperature drop and higher plasma histamine levels Higher number of mast cells in the tissues of the 129ScEvBrd mice compared to BALB/c animals No differences in Ova-specific IgE or IgG 1 levels or mast cell degranulation efficiency between both strains	
[57]	C57BL/6	4 × 1 mg peanut protein + 10 µg cholera toxin	1 × 5 mg peanut protein, i.p.	Temperature drop, systemic release of histamine and Cys-leukotriene Local swelling of nose and mouth Reduced activity Increased production of allergen-specific IgG1 and IgE antibodies Early mast cell activation, followed by infiltration of neutrophils, lymphocytes, and eosinophils into the peritoneum	
[40]	C3H/HeJ	2 × 5 or 25 mg peanut protein + 10 µg cholera toxin, i.g.	1 × 10 mg peanut extract, i.g.	Only the lower allergen dose (5 mg) was able to successfully induce allergic sensitization Robust peanut-specific IgE production Mast cell degranulation & plasma histamine release	In this publication Li et al. defined a symptom score for the evaluation of the induced allergic reactions (see Table 2) which has proven very useful for many other mouse models.
[72]	BALB/c	2 × 50 µg Ova plus 1 mg Alum, i.p.	Continuous challenge by feeding of Ova-containing food pellets for a period of seven days	Weight loss and temperature drop Production of Ova-specific IgE antibodies Pronounced diarrhea and inflammation of the small intestine	
[50]	BALB/c	6 × 20 µg Pru p 3 + 20 ng LPS, i.n.	1 × 100 µg Pru p 3, i.p.	Anaphylactic reactions (temperature drop, inactivity, and increased respiratory rates) Increased production of Pru p 3-specific IgE and IgG1 antibodies Increased production of IL-4 and IFN-γ, but reduced IL-10 production from splenocytes	Only available Pru p 3 allergy model
[48]	B10.A	1 × 100 µg + 5x 50 µg wheat gliadin fraction + 4 mg Alum, i.p.	1 × 10 mg gliadin, i.g. + 30 min of strenuous physical treadmill exercise (15 m/min, 20 % gradient)	Production of gliadin-specific IgE antibodies Anaphylactic reactions (temperature drop and significantly lower activity levels)	Mouse model of exercise induced anaphylaxis.
**Mouse Models using Genetically-modified Mouse Strains**					
**Reference**	**Mouse Strain**	**Sensitization**	**Challenge**	**Read-Out**	**Comments**
[73]	IL-4RaF709	12 × 5 mg Ova + 20 µg cholera toxin, i.g. OR 12 × 5 mg Ova without adjuvant, i.g.	1 × 150 mg Ova, i.g.	Significantly increased basal IgE production in naive IL-4RaF709 animals Temperature drop, diarrhea, and systemic mast cell activation Production of ovalbumin-specific IgE antibodies increased allergen-specific IgE levels if the animals were sensitized with Ova plus cholera toxin	Due to the Th2-promoting milieu IL-4raF709 can be sensitized to allergen without adjuvant
[74]	Foxp3 CNS1^−/−^	Not performed	Not performed	Foxp3 CNS1^−/−^ mice with distinct Th2-mediated pathologies at both the gastrointestinal tract and the lung characteristic of both allergic inflammation and asthma Decreased body weight likely caused by gastritis and plasmacytic enteritis with increased frequencies of plasma cells in the intestinal lamina propria and crypt abscesses Spontaneous development of Th2-biassed responses (enhanced production of IL-4, IL-5, and IL-13 by GATA3^+^CD4^+^ T cells, constitutively increased serum levels of IgE and IgA specific for both autoantigens, as well as food antigens Alterations in gut microbiome (decreased ratios of Firmicutes to Bacteroidetes)	Th1 and Th17 responses unchanged No multiorgan autoimmune responses observed
[75]	WAS^−/−^	Not performed	Not performed	WAS^−/−^ mice develop T cell dependent, severe progressive colitis (infiltration of neutrophils and lymphocytes, enhanced levels of IFN-γ, IL-4, and IL-13) by six months of age (50% fatality rate Only neutralization of IL-4, but not IFN-y, was able to abrogate the development of colitis	
[76]	WAS^−/−^			WAS^−/−^ mice develop spontaneous allergic sensitization to common food allergens, most pronounced for wheat and soy, paralleled by intestinal mast cell expansion Th2-mediated intestinal inflammation was even further enhanced when mice were conditionally deleted for WASP in Foxp3^+^ Tregs	Mechanistically, WASP-deficiency drives Th2 responses by interfering with normal Treg function
[77]	WAS^−/−^	7 × 5 mg Ova without adjuvant, i.g., v.s. 3 × 50 µg Ova + 100 µL Alum, i.p.	1 × 50 mg Ova	WAS^−/−^ mice sensitized without adjuvant developed significantly lower Ova-specific IgE levels compared to classical Ova/alum sensitization Upon oral challenge Ova 79% of WAS^−/−^ mice exhibited symptoms of systemic intestinal anaphylaxis, (elevated serum levels of mMCPT1, mortality rates > 50%)	WAS^−/−^ mice mimic both polysensitization and highly bioactive, but low-level IgE production frequently observed in human food allergic patients
**Humanized Mouse Models**					
**Reference**	**Mouse Strain**	**Humanization & Sensitization**	**Challenge**	**Read-Out**	**Comments**
[78]	Mice expressing the α-chain of human FcεRI in a CD11c-restricted manner	2 × 100 µg Ova mixed 1:1 with Alum, i.p.	3–6 × 50 mg Ova, i.g.	Mice with CD11c-restricted expression of the human FcεRI α-chain had lower levels of gastro-intestinal inflammation (lower levels of mast cell transcripts, lower production of IL-4, IL-13, CCL-2, and IL-6, lower systemic levels of mMCP1) compared to wildtype control mice	IgE-signaling in human DCs is involved in down-regulating allergic mucosal inflammation
[79]	NOD-SCID-γc^−/−^	I.p. injection of 2 × 10^7^ PBMC from allergic donors with high allergen-specific IgE levels in combination with the respective allergen (20 µg) followed by i.p. boost with 20 µg allergen 8 days later	1 × 20 µg allergen rectally or 50 µg allergen orally (d21)	2–6 h after challenge, assessment of allergen-specific induction of gastro-intestinal inflammation via mini-endoscopy, histology Production of allergen-specific IgE Allergen-specific T cell proliferation and cytokine production	Blocking experiments demonstrated that gut inflammation in this model was mediated by human IgE
[80]	NOG IL-3/GM-Tg mice	1 × 10^5^ or 4 × 10^4^ human umbilical cord blood-derived CD34^+^ hematopoietic stem cells i.v. 24 h after irradiation	Not performed	Significant increase in human total myeloid cells (CD33^+^), granulocytes (CD66b^+^), and monocytes (CD14^+^) in peripheral blood Human eosinophils, basophils, neutrophils, and mast cells detectable 18 weeks after transplantation Activation and IgE-mediated passive cutaneous anaphylaxis reactions by human basophils and mast cells	
[81]	NOD-SCID-γc^−/−^ stem cell factor (SCF)-Tg mice	5 × 10^4^–10^5^ human CD34^+^ cord blood stem cells, i.v., 6 weeks later sensitization: 8 × 22.5 mg peanut butter without adjuvant, i.g.	1 × 350 mg peanut butter, i.g.	Human Th1- and Th2-effector cells, Foxp3^+^CD127^low^CD25^+^ Tregs, and CD19^+^HLA-DR^+^ B cells detectable in spleen and jejunum of the engrafted animals Production of human peanut-specific IgE antibodies and IgE-dependent activation of human mast cells resulting in anaphylactic reactions (temperature drop, tryptase release)	Humanized mouse models can be used to recreate the multistep and highly complex human allergic responses
[82]	NSG	3 × 10^7^ PBMC from peanut allergic individuals mixed with 100 µg peanut extract (i.p. split in 2 injections sites), followed by weekly i.p. injections (4×) with 100 µg peanut extract	4 × weekly 300 µg peanut extract, i.g.	Production of human peanut-specific IgE Induction of allergic symptoms in peanut allergic mice (anaphylaxis score, locomotor activity)∙ Histamine level in plasma	Humanized mouse model was used to compare the effectiveness of an experimental adeno-associated virus (AAV)-based expression of anti-human IgE to the already established anti-human IgE treatment with Omalizumab

**Table 2:** Symptom score after [40].

Score	Symptoms
0	No symptoms
1	Scratching and rubbing around nose and head
2	Puffiness around eyes and mouth, diarrhea, pillar erect, reduced activity, and/or decreased activity with increased respiratory rate
3	Wheezing, labored respiration, and cyanosis around mouth and tail
4	No activity after prodding or tremor and convulsion
5	Death

## Abbreviations

AAV	Adeno-associated virus
AIT	Allergen-specific immunotherapy
Alum	Aluminum hydroxide
APC	Antigen presenting cell
cAMP	Cyclic adenosine monophosphate
CT	Cholera toxin
CNS1	Non-coding DNA sequence 1
DC	Dendritic cell
DAMP	Damage associated molecular pattern
GATA3	GATA binding protein 3
GM-CSF	Granulocyte-macrophage colony-stimulating factor
HSC	Hematopoietic stem cells
IL(-25)	Interleukin (25)
ILC2s	Innate like lymphocytes type II cells
iTregs	Induced Tregs
LPS	Lipopolysaccharide
MAPK	Mitogen-activated protein kinase
mMCP1	Mouse mast cell protease 1
NFκB	Nuclear factor “kapa-light-enhancer“ of activated B cells
NOD	Non-obese diabetic
Ova	Ovalbumin
PBMC	Peripheral blood mononuclear cells
SCID	Severe combined immunodeficiency
SCF	Stem cell factor
SEB	Staphylococcus enterotoxin B
TCR	T cell receptor
Th1/2/17	T helper 1/2/17 cell
TLR(4)	“Toll“-like receptor (4)
(i)Treg	(Peripherally induced) regulatory T cell
TSLP	Thymic stromal lymphopoietin
WAS(P)	Wiskott-Aldrich syndrome (protein)
